# Hepatitis A Vaccine

**DOI:** 10.1155/S1064744995000214

**Published:** 1995

**Authors:** Sebastian Faro


Infectious Diseases in Obstetrics and Gynecology 3:1–2 (1995)

© 1995 Wiley-Liss, Inc.

### Hepatitis A Vaccine

Hepatitis A is a significant infectious disease that occurs worldwide. The ease of travel has facilitated the transmission of this disease, thereby increasing the number of individuals at risk. Most hepatitis A infections resolve without serious sequelae, and a good number of infected individuals are asymptomatic. Although most symptomatic infections are self limiting and are not responsible for any significant morbidity or mortality, this infection does result in a considerable economic burden, with an estimated cost of $200 million annually.

The most logical approach to the management of this disease is prevention because no treatment currently exists. The transmission of this virus occurs essentially by the fecal-oral route. Therefore, prevention has focused on avoiding exposure and promoting hygienic measures. An important preventive measure as well as partial treatment is the use of human immune globulin, which contains antibodies against the hepatitis A virus.

The successful development of a vaccine could lead to the eradication of the hepatitis A virus because the human is the only natural reservoir. This vaccine is prepared by treating the virus with formaldehyde, which produces an inactivated virus without destroying its antigenic character, thereby allowing the recipient of the vaccine to manifest an antibody response. In several studies, the vaccine has proven to be safe, well tolerated, and immunogenic in all groups tested. When the vaccine has been administered to adults, the vaccine-induced antibodies have been present for over one year. When a booster dose has been administered 6–12 months after the initial vaccination, extremely high antibody titers have been produced. Moreover, it has been mathematically determined that the administration of a booster vaccination has the potential of producing protective antibodies for more than 20 years.

Discussions will likely continue regarding which groups should receive the vaccine. The first group recommended to receive the inactivated hepatitis A vaccine includes children. Other groups that have been designated to receive the vaccine are the military, homosexual men, injection-drug users, day-care workers, health-care workers, sewage-treatment workers, and food handlers. However, this strategy of designating selected groups to receive vaccinations has not proven effective in administering the hepatitis B vaccine. Administering the hepatitis B vaccine to only high-risk groups has not resulted in any significant reduction in the frequency of hepatitis B infections.

Once again, pregnant women have not been designated to receive the vaccine. Why not develop a strategy to vaccinate all those who do not have antibodies against hepatitis A? Included in this strategy should be adolescents, a group proven to be extremely sexually active. Screening could be accomplished at STD clinics, family-planning clinics, and Planned Parenthood clinics at the time of preconceptual counseling and at the first prenatal visit. The same strategy would apply to the varicella vaccine soon to be released. No doubt, vaccines developed for frequently

Editorial

Received April 25, 1995
Accepted April 26, 1995

occurring diseases that have no specific treatment should be administered to the population that lacks antibodies to that specific agent. The wide distribution of a vaccine is the only way to eradicate a specific disease, for example, smallpox.

*Sebastian Faro, M.D., Ph.D.*
*Editor-in-Chief*

